# Functional decline in nursing home residents: A prognostic study

**DOI:** 10.1371/journal.pone.0177353

**Published:** 2017-05-11

**Authors:** Javier Jerez-Roig, Lidiane Maria de Brito Macedo Ferreira, José Rodolfo Torres de Araújo, Kenio Costa Lima

**Affiliations:** 1 Postgraduate Program in Collective Health, Odontology Department, Federal University of Rio Grande do Norte (UFRN), Avenida Salgado Filho 1787, Lagoa Nova, Natal-RN, Brazil; 2 Can Misses Hospital, Ibiza, Spain; 3 Postgraduate Program in Health Sciences, Center of Health Sciences, Federal University of Rio Grande do Norte (UFRN), Av. Gustavo Cordeiro de Farias, s/n, Petrópolis, Natal RN, Natal-RN, Brazil; Banner Alzheimer's Institute, UNITED STATES

## Abstract

**Aim:**

To verify the probability of maintaining functional capacity in basic activities of daily living and identify the prognostic factors of functional decline in institutionalized older adults.

**Methods:**

A longitudinal study is presented herein, with 5 waves every 6 months, throughout 2 years (2013–2015), conducted with individuals ≥60 years old in 10 nursing homes in the city of Natal-RN (Brazil). Functional capacity was assessed by the items ‘eating’, ‘personal hygiene’, ‘dressing’, ‘bathing’, ‘transferring’, ‘toileting’ and ‘walking’, through a 5-item Likert scale. Sociodemographic, institution-related and health-related variables were considered to establish the baseline. Time dependent variables included continence decline, cognitive decline, increase in the number of medication, and incidences of falls, hospitalizations and fractures. The actuarial method, the log-rank test and Cox's regression were applied as statistical methods.

**Results:**

The cumulative probability of functional maintenance was 78.2% (CI 95%: 72.8–82.7%), 65.1% (CI 95%: 58.9–70.5%), 53.5% (CI 95%: 47.2–59.5%) and 44.0% (CI 95%: 37.7–50.2%) at 6, 12, 18 and 24 months, respectively. Predicting factors for functional decline were: severe cognitive impairment (HR = 1.96; p = 0.001), continence decline (HR = 1.85; p = 0.002) and incidence of hospitalizations (HR = 1.62; p = 0.020), adjusted by the incidence of depression, age, education level, presence of chronic diseases and low weight.

**Conclusions:**

The cumulative probability of maintaining functional capacity in institutionalized older adults was only 44% at the 2-year follow-up. Prognostic factors for functional decline included severe cognitive impairment, continence decline and incidence of hospitalizations.

## Introduction

Reductions in fecundity and mortality rates, along with scientific and technological advances, have enabled the increase in life expectancy. As a consequence, population aging is a common phenomenon in most modern societies, and it is estimated that in 2050 the number of older adults in the world can reach 2 billion [1,2]. In developing countries such as Brazil this phenomenon has occurred in an accelerated manner; there was a 700% increase in people aged 60 and older during the last five decades and projections show that the aging index will increase from 36 in 2016 to 76 in 2030 [3]. However, 70% of Brazilian municipalities lack nursing homes (NHs), the majority of the existing institutions are private or not for profit (public NHs are scarce), and the country institutionalization rate is only 1% [4].

In this context, a series of challenges emerge for public health, such as maintenance of quality of life and autonomy, and delaying the onset of impairment processes in this increasing aging population, among other challenges [5]. Disabilities or impairments have high socioeconomic impact that is associated with institutionalization and death, frequently generating long term care and complications throughout time [6]. Functional capacity can change/deteriorate considerably fast and, therefore, the study of transitions in the process of functional decline is fundamental for the planning of health services [7]. Longitudinal studies are the most adequate type of epidemiological studies to understand better the impairment processes that occur during senility. Only longitudinal studies are capable of determining the effects of several factors on functionality, as well the magnitude and direction of the association between this outcome and the other independent variables [8].

There are few existing longitudinal studies on the dynamics of functional decline, that were conducted in the U.S., Switzerland and China [9–14]. There is a lack of studies carried out in Latin America and no previous works have systematically applied more than two waves to the cohort to analyze the detailed evolution of functioning. The study presented herein aims at broadening knowledge on functional decline in NH, through a 5-wave prospective study applied to a representative sample in a Northeast Brazil capital. The objective of this study was to verify the probability of maintaining functional capacity in Basic Activities of Daily Life (BADL) and identify the prognostic factors for functional decline in institutionalized older adults.

## Materials and methods

### Design

A longitudinal, prospective study is presented herein, carried out in 10 (71.4%) of the 14 NHs registered in the Sanitary Vigilance of the municipality of Natal-RN (Brazil), throughout a 24-month period (2013–2015). Power analysis within the calculation of sample size utilized the association between the outcome (functional decline) and the independent variable 'cognitive state'. The following statistical parameters were considered: 48.6% proportion of cases among the exposed individuals, 27.8% proportion of cases among the non-exposed individuals, 1.78 relative risk, significance level of 0.05 and power of 0.80. The analysis indicated a minimum sample of 164 individuals; when considering 25% attrition, the final sample should be 205 subjects.

### Subjects

All residents at least 60 years old that were registered in the institutions during the study period were included. This age group is characterized by the World Health Organization (WHO) as 'older adults' in developing countries [15]. Exclusion criteria considered older adults that had total functional impairment for BADL, and those in a terminal state, coma or under palliative care. Further information is available in Jerez-Roig et al. (2016) [16].

### Measures

The dependent variable of the study was the presence of functional decline for BADL using the Barthel's index [17]. The total functional decline score consisted of 7 items (‘eating’, ‘personal hygiene’, ‘dressing’, ‘bathing’, ‘transferring’, ‘toileting’ and ‘walking’) with Likert-type, 5-point response options that were summed across the 7 items. The scale ranged from 0 (total limitation in all BADL) to 28 (no limitations). For those individuals with increasing and decreasing scores throughout the study period, the presence of functional decline was considered when the score was lower than the initial score.

Also, sociodemographic, institution- and health-related variables were considered, which are detailed in Jerez-Roig et al. (2016)[16].Time-dependent variables included the incidence of hospitalizations, depression, falls and fractures, as well as continence and cognitive decline during the period. Independent variables are depicted in Table 1.

**Table 1:** Independent variables included in the study of functional decline in institutionalized older adults in Natal/RN. Natal/RN, 2017.

Variables	Categories	Assessment
Sociodemographic variables		
Age	60–81 ≥82	Chart review. According to birth date; in years.
Sex	Men Women	Chart review.
Education level	Illiterate, literate, fundamental I and II High school and undergraduate	Rated by the participant (when cognitive impairment not present), tutor or NH personnel (director, nursing assistant).
Marital status	Married Other (single, divorced or widow/er)	Rated by the participant (when cognitive impairment not present), tutor or NH personnel (director, nursing assistant).
Race	White Other (brown, afro-american, yellow or indigenous)	Rated by the research team.
Private health plan	No Yes	Chart review and/or rated by the NH director.
Institution-related variables		
Type of NH	Not for profit For profit	Rated by the NH director.
Reason for institutionalization	No caregiver Lived alone No home Disease Own choice Unemployed Other reasons	Rated by the participant (when cognitive impairment not present), tutor or NH personnel (director, nursing assistant).
Institutionalization time	1–39 ≥40	Chart review. According to the date of admission in the institution; in months.
Number of residents/caregiver	0–8.0 ≥8.5	Rated by the NH director, according to the number of caregivers per resident in a common morning shift.
Health-related variables		
Smoking	No Yes (current or former smoker)	Rated by the NH personnel (director, nursing assistant).
Alcohol (current use)	No Yes	Rated by the NH personnel (director, nursing assistant).
Physical activity	No Yes	Rated by the NH personnel (director, nursing assistant).
Medication	No Yes (1 or more daily drugs)	Chart review.
Types of medication	A (alimentary tract and metabolism) B (blood and blood forming organs) C (cardiovascular system) D (dermatologicals) G (genitourinary system and sex hormones) H (systemic hormonal preparations) J (antiinfectives for systemic use) L (antineoplastic and immunomodulating agents) M (musculo-skeletal system) N (nervous system) R (respiratory system) S (sensory organs) V (various)	According to ATC/DDD classification system (2013).
Chronic diseases	Arterial hypertension Diabetes Dementia Parkinson’s disease Mental disease Osteoporosis Depression Dyslipidemia Stroke Cancer Pulmonary disease Rheumatic disease Kidney failure	Chart review. Diagnosed conditions.
Chronic disease	No Yes (1 or more of the above-mentioned)	Chart review. Diagnosed conditions.
Cognitive state	Intact, mild or moderate impairment Severe cognitive impairment	Assessed by the research team applying the Pfeiffer’s test to the resident.
Mobility state	Gait without aid Gait with aid, wheelchair or bedridden	Rated by the NH personnel (nursing assistant), according to the observed ability.
Urinary incontinence	No Yes	The research team interviewed the nursing assistants using the Minimum Data Set 3.0.
Fecal incontinence	No Yes	The research team interviewed the nursing assistants using the Minimum Data Set 3.0.

Regarding the types of medication, the international ATC/DDD (*Anatomical Therapeutic Chemical classification system and the Defined Daily Dose*) classification system was applied for year 2013, as recommended by the WHO for studies on medication use [18]. Only daily drugs were considered; eye drops, inhalators, vitamins and minerals were included, while nutritional support, ointments and systemic antibacterial drugs (type J) used during a period of time inferior to 30 days were excluded. This information, along with other health-related information, were obtained from medical records or were provided by the older adults or personnel at the institutions, in the case of cognitive impairment.

Regarding mobility evaluation, the following states were considered, according to nursing assistant criteria (self-rated) and the main researcher (first author) observation: walks without aid, walks with aid, uses wheelchair, and bedridden. The cognitive state was evaluated by Pfeiffer's Test for the baseline (wave 1), and after 1 and 2 years (waves 3 and 5, respectively). This test evaluates short- and long- term memory, orientation, information on daily activities, and mathematical capacity. This instrument enables the classification of older adults in intact mental function, and slight, moderate or severe cognitive decline, taking into consideration the education level of the individual [19].

For the nutritional state, the Mini Nutritional Assessment (MNA) was applied by previously trained and calibrated researchers, who classifies the older adults in three different groups: individuals with adequate nutritional state (MNA ≥ 24), risk of malnutrition (MNA between 17 and 23.5); and malnutrition (MNA < 17)[20]. The Body Mass Index (BMI) was calculated from the relationship between weight (in kg) and squared height (in meters), using an electronic Tanita® scale and the average of two measurements, taken with an exact-height (1mm precision) portable stadiometer. The following categories were considered: underweight (<22 kg/m^2^), eutrophic (≥ 22 and< 27 kg/m^2^) and overweight (≥ 27 kg/m^2^)[21].

### Procedures

This study is part of the research project "Human aging and health: the reality of institutionalized older adults in the city of Natal/RN", approved by The Research Ethics Committee of the Federal University of Rio Grande do Norte, under protocol number 013/2014. The NH directors participating in the study signed an agreement. The main researcher interviewed the assistant nurses and caregivers, who provided the necessary information regarding the Barthel index. Informed consent forms were signed by the older adults or legal tutor/guardian and their nursing assistants. These ethical procedures follow the National Health Council in resolution 196/96.

### Data analysis

The actuarial method was utilized to analyze functional decline throughout the 5-wave cohort. Log-rank was applied for bivariate analysis. Those variables with *p*<0.25 and variables "age" and "sex" were considered susceptible for testing in the multiple model. Multivariate analysis was developed by Cox's regression, using a 0.05 significance level. Forward selection was utilized to introduce covariables in the model, firstly introducing those variables with higher hazard ratio (HR) values, and observing the behavior and adjustment of the model (stepwise forward). Risk measurements were presented for HR, with the respective confidence intervals (CI) and *p* values. Finally, the proportionality test was carried out for the final model, followed by Schoenfeld residual analysis to verify validity of Cox's semiparametric model. The software STATA version 12 was utilized.

## Results

The final total sample was constituted of 280 older adults, mostly females (75.4%), with average age 80.4 years (SD = 8.8). Regarding functional capacity, 93 older adults (33.2%) were totally independent and 187 were semidependent (66.8%), *i*.*e*., presented limitations in at least one BADL. Further information on the sampling process is available in Jerez-Roig et al. (2016).^14^

**Fig 1:**
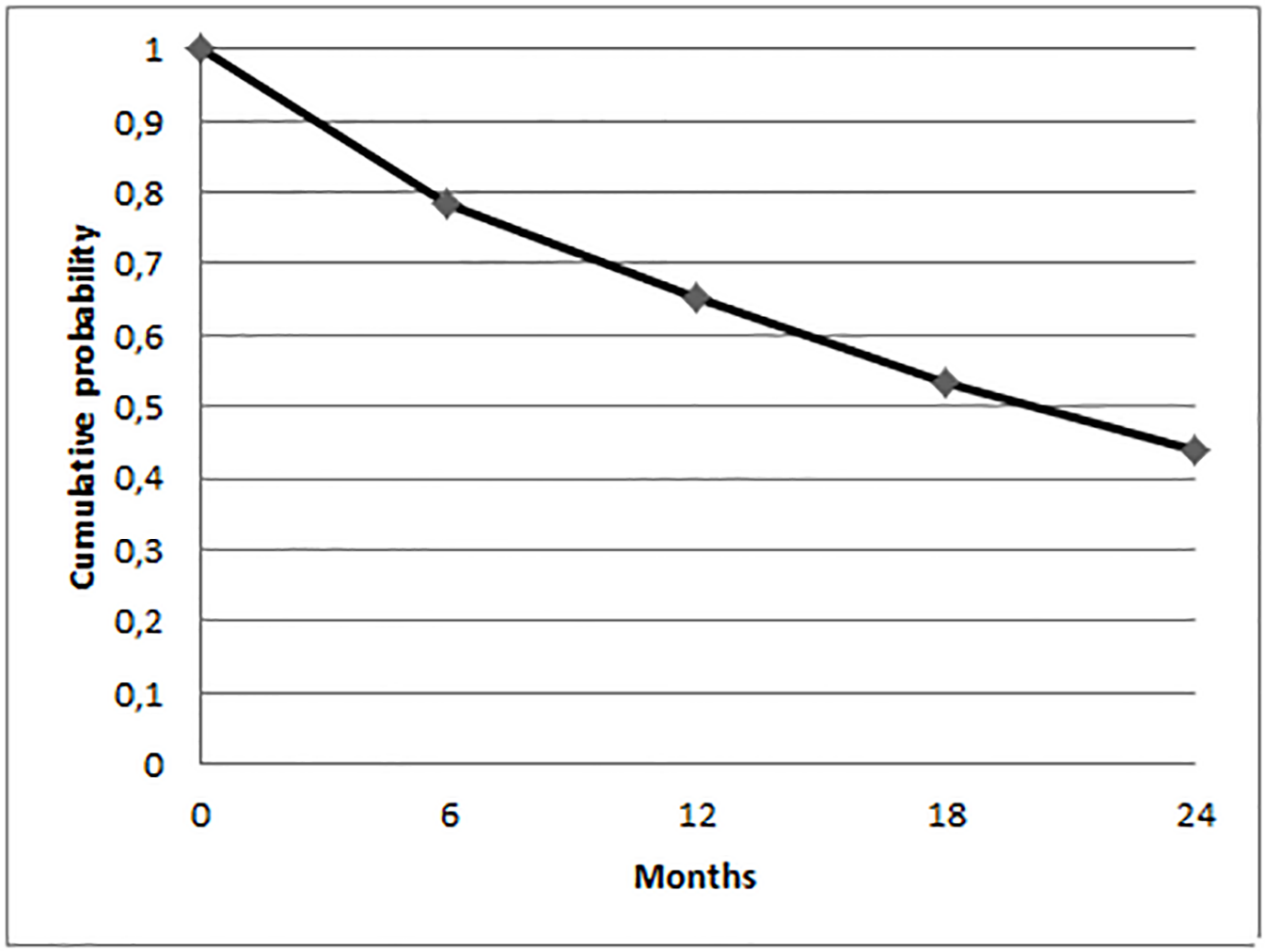
Cumulative probability of maintaining functional capacity in institutionalized older people (n = 280) in the city of Natal/RN (Brazil), during the period 2013–2015. Natal/RN (Brazil), 2016.

Table 2 contains the complete results for the life table analysis. The cumulative probability of maintaining functional capacity was 78.2% (CI 95%: 72.8–82.7%), 65.1% (CI 95%: 58.9–70.5%), 53.5% (CI 95%: 47.2–59.5%) and 44.0% (CI 95%: 37.7–50.2%) at 6, 12, 18 and 24 months, respectively. Fig 1 shows the survival curve, more pronounced at the beginning and smoother afterwards.

**Table 2:** Probability of maintaining functional capacity by the actuarial method (incident cases of functional decline in institutionalized older adults in Natal/RN). Natal/RN, 2016.

Interval (months)	Initially exposed	Functional decline cases	Number of observation losses	Exposed to functional decline	Conditional probability of functional decline	Conditional probability of maintaining functional capacity	Cumulative probability of maintaining functional capacity
0├ 6	280	59	18	271	0.2177	0.7823	0.7823
6├ 12	203	33	14	196	0.1683	0.8317	0.6506
12├ 18	156	27	7	152.5	0.1770	0.8230	0.5354
18├ 24	122	21	8	118	0.1779	0.8221	0.4401

According to log-rank, there were statistically significant differences (*p*<0.05) in the survival curves (maintenance of functional capacity) for the variables education level, private health plan, type of institution, institutionalization time, use of medication, type H medication, type N medication, nutritional state, consumption of alcohol, Parkinson's disease, osteoporosis, kidney failure, initial functional impairment, severe cognitive impairment, immobility, urinary and fecal incontinences, incidences of hospitalizations and fractures, as well as decline of urinary/fecal continence. Survival rates for the covariables with *p*<0.10 are depicted in Table 3. The remaining independent variables, except sex, are not shown.

**Table 3:** Survival rates for maintenance time of functional capacity in relation to the covariables with *p*<0.10, besides sex, in institutionalized older adults (n = 280) in the city of Natal/RN. Natal/RN, 2016.

	n	Functional decline	Maintenance of functional capacity (% and CI 95%)	*p* (*log-rank*)
Age				
60–81	140	67	41.4 (31.4–51.2)	0.067
≥82	140	73	34.2 (24.2–44.4)	
Sex				
Men	69	31	36.5 (21.6–51.5)	0.459
Women	211	109	38.1 (30.1–46.0)	
Educationlevel				
High school- Undergraduate	80	46	29.9 (18.1–42.7)	0.030*
Illiterate-Fundamental II	160	71	43.8 (34.0–53.2)	
Private health plan				
Yes	105	60	28.6 (18.1–40.1)	0.007*
No	174	80	42.7 (33.4–51.6)	
Type of institution				
For profit NH	102	58	27.3 (16.7–39.1)	0.007*
Not for profit NH	178	82	43.5 (34.4–52.2)	
Number of residents/caregiver				
0–8.0	155	69	40.8 (30.7–50.6)	0.060
≥8.5	125	71	34.3 (24.5–44.3)	
Institutionalization time				
1–39 months	140	76	32.6 (23.0–42.6)	0.029*
≥40 months	139	64	42.8 (32.5–52.7)	
Medication				
No	13	2	84.6 (51.2–95.9)	0.010*
Yes	267	138	35.3 (28.2–42.6)	
Type H: thyroid medication				
No	252	124	39.7 (32.2–47.1)	0.036*
Yes	25	16	16.3 (3.2–38.6)	
Type N: medication for the nervous system				
No	60	21	57.8 (41.6–70.9)	0.003*
Yes	217	119	32.1 (24.4–40.0)	
Nutritional state				
Eutrophy	104	44	45.3 (33.0–56.8)	0.006*
Risk of malnutrition/malnutrition	159	89	35.2 (26.4–44.1)	
Smoking				
No	193	102	34.6 (26.2–43.0)	0.058
Yes	86	37	45.9 (32.6–58.2)	
Alcohol				
No	268	137	36.3 (29.1–43.5)	0.032*
Yes	11	2	80.0 (40.9–94.6)	
Lung disease				
No	268	138	36.7 (29.5–43.8)	0.099
Yes	12	2	80.9 (42.9–94.9)	
Parkinson's disease				
No	270	132	39.5 (32.2–46.7)	0.009*
Yes	10	8	0	
Osteoporosis				
No	250	130	35.5 (28.1–42.9)	0.035*
Yes	30	10	56.9 (32.4–75.5)	
Kidney failure				
No	270	133	38.6 (31.3–45.8)	0.043*
Yes	10	7	13.0 (0.7–43.3)	
Initial functional impairment				
No	93	37	49.3 (36.3–61.0)	<0.001*
Yes	187	103	31.8 (23.5–40.4)	
Cognitive state				
Intact/Slight- or moderate impairment	130	52	48.6 (37.5–58.8)	<0.001*
Severe cognitive impairment	145	85	27.8 (19.0–37.3)	
Mobility state				
Walks without aid	136	64	41.6 (31.3–51.5)	0.027*
Walks with help/Uses wheelchair/Bedridden	144	o76	34.4 (24.7–44.4)	
Urinary incontinence				
No	131	55	45.7 (34.7–56.1)	0.001*
Yes	149	85	30.6 (21.6–40.1)	
Fecal incontinence				
No	192	88	41.3 (32.4–49.9)	0.007*
Yes	88	52	30.2 (19.0–42.2)	
Falls				
No	263	135	36.7 (29.4–44.0)	0.070
Yes	17	5	55.4 (21.1–79.9)	
Incidence of hospitalizations				
No	209	86	45.5 (36.6–54.0)	<0.001*
Yes	71	54	18.4 (9.4–29.8)	
Incidence of fractures				
No	262	125	39.7 (32.2–47.1)	0.002*
Yes	18	15	13.3 (2.0–35.3)	
Decline of continence (urinary and/or fecal)				
No	190	80	47.7 (38.7–56.1)	<0.001*
Yes	90	60	17.3 (8.6–28.4)	

Table 4 contains the results of Cox univariate analysis for sociodemographic, institution- and health-related variables with *p* values under 0.10 that were not part of the multivariate model.

**Table 4:** Univariate analysis of covariables sex, as well as sociodemographic, related to the institution and health state variables with *p*< 0.10 that were not part of the multivariate model, regarding functional decline in institutionalized older adults (n = 280) in Natal/RN. Natal/RN, 2016.

	HR	CI 95%	*p* (Cox)
Sex			
Men	ref		0.504
Women	1.14	0.77–1.71	
Type of institution			
For profit NH	ref		0.015*
Not for profit NH	0.66	0.47–0.92	
Number of older adults/caregiver			
0–8.0	ref		0.090
≥8,5	1.33	0.96–1.85	
Institutionalization time			
1–39 months	ref		0.050
≥40 months	0.72	0.51–1.00	
Medication			
No	ref		0.033*
Yes	4.57	1.13–18.46	
Type H: for the thyroid			
No	ref		0.061
Yes	1.65	0.98–2.78	
Type N: for the nervous system			
No	ref		0.007*
Yes	1,88	1,18–3,00	
Nutritional state			
Eutrophy	ref		0.013*
Risk of malnutrition/malnutrition	1.58	1.10–2.27	
Alcohol			
No	ref		0.069
Yes	0,27	0,07–1,11	
Parkinson’s disease			
No	ref		0.023*
Yes	2.30	1.12–4.71	
Kidney failure			
No	ref		0.075
Yes	2.00	0.93–4.30	
Osteoporosis			
No	ref		0.060
Yes	0.54	0.28–1.03	
Initial functional impairment			
No	ref		0.003*
Yes	1.79	1.22–2.61	
Mobility state			
Walks without aid	ref		0.047*
Walks with help/Uses wheelchair/Bedridden	1.40	1.00–1.96	
Urinary incontinence			
No	ref		0.004*
Yes	1.64	1.17–2.30	
Fecal incontinence			
No	ref		0.015*
Yes	1.53	1.09–2.16	
Incidence of fractures			
No	ref		0.007*
Yes	2.08	1.21–3.56	

Table 5 shows the results of multivariate analysis. Severe cognitive impairment, along with urinary and/or fecal incontinence and hospitalizations during the study period were identified as risk factors for the occurrence of functional decline, independently from the incidence of depression, Parkinson's disease, kidney failure, education level, reason for institutionalization "by own choice", smoking habits, marital status, private health plan and low weight. The older adults that presented severe cognitive impairment at baseline were 96% more at risk of functional decline for BADL. The older adults that suffered deterioration of continence (urinary and/or fecal) and were hospitalized during the period presented 85% and 62% more risks, respectively, of functional decline in BADL. The proportionality test for risk demonstrated the global proportionality of the final model (*p* = 0.785).

**Table 5:** Final model for functional decline in institutionalized older adults (n = 280) in Natal/RN, according to Cox's model. Natal/RN, 2016.

	HR	AdjustedHR	CI 95%	*p*
Cognitive state				
Intact/moderate cognitive impairment	ref			0.001*
Severe cognitive impairment	1.91	1.96	1.30–2.94	
Decline of continence				
No	ref			0.002*
Yes	1.99	1.85	1.24–2.75	
Incidence of hospitalization				
No	ref			0.020*
Yes	2.18	1.62	1.08–2.43	
Incidence of depression				
No	ref			0.069
Yes	1.42	1.69	0.96–2.97	
Age				
60–75	ref			0.316
≥76	1.59	1.25	0.81–1.93	
Education level				
High school/undergraduate	ref			0.452
Illiterate/Fundamental II	0.69	0.86	0.57–1.28	
Chronic diseases				
No	ref			0.462
Yes	1.50	1.26	0.68–2.32	
Low weight (BMI)				
No	ref	1.05		0.808
Yes	1.22		0.71–1.56	

## Discussion

The results presented herein indicate that the probability of not suffering functional decline in institutionalized older adults, initially independent and semi-dependent, dropped to only 44% after 24 months. It also was observed that the decline process of functional capacity was progressive and presented an exponential form, with more accelerated decline at the beginning, which was progressively less pronounced towards the end.

The incidence of functional decline in this sample was approximately 54%. Also, 33% maintained unaltered functional capacity during the period and only 14% presented functional improvement in one or more assessments [16]. After systematic reviews and to the best of the authors' knowledge, there are no studies performing life table analysis in a sample of institutionalized older adults, which prevents comparisons with the results herein obtained.

The Cox model verified that severe cognitive impairment was the strongest risk factor for functional decline in this sample of institutionalized older adults. It must be highlighted that approximately 50% of this sample presented severe cognitive impairment at baseline and this subgroup of residents tends to show the greatest deterioration in 'eating', a late loss BADL, which was the most affected task [11,16]. Cognitive deficits are also significant factors in health decline, even when there are no association with physical frailty [9]. Cognitive performance is fundamental for the execution of daily life activities, and a systematic review identified cognitive impairment as one of the most important risk factors for functional status decline in community-dwelling older adults [22].

In institutionalized individuals, the association between functional decline and cognition has also been well-documented in several studies [9–11,13]. A longitudinal study conducted in NH residents verified that baseline cognitive performance has stronger effects than the diagnosis of diseases, leading to increases in functional impairment over time [9]. In the same line, another U.S. study also verified that, upon admission, cognitive impairment was the most important predictor of BADL dependence and the severity of impairment influenced the severity of functional decline over time [13]. Also, a survival study carried out by Burge *et al*. in Swiss NH identified the same association and confirmed that individuals with impaired cognition were less likely to experience improvements in BADL performance [10].

Decline in bladder and/or bowel continence during the study period was also a significant predicting factor for functional deterioration in the study presented herein. Incontinence is a multifactorial syndrome, that is part of the disabling senility process and can lead to a decrease in physical capacity and worse BADL performance [23,24]. A previous cross-sectional study identified a high prevalence of functional urinary incontinence (due to cognitive and/or physical limitations) at the baseline of this study, and also a strong association between physical limitations and immobility, and between physical inactivity and cognitive impairment [24]. Three longitudinal studies in different countries also have identified fecal and/or urinary incontinence as significant risk factors for functional decline in institutionalized older adults [10,12,14]. Besides, Burge *et al*. found that sphincter control was a predicting factor for functional improvement [10].

The third risk factor for functional decline that was included in the final model was the incidence of hospitalizations during the 2-year period. The inverse direction of this association is more explored in scientific literature, and a recent systematic review found moderate evidence that disability is associated with future NH admission and hospitalization [25]. Also, it is well known that many NH residents experience illness episodes that require brief hospital stays, during which many suffer physical deconditioning, leading to long-term loss of independence and quality of life [13,26]. Therefore, it is fundamental to intensify rehabilitation interventions during hospital stays, as the early introduction of transitional care programs have proved to be effective in improving functional status in hospitalized, at-risk older adults [10,26].

Nevertheless, sociodemographic variables such as age and the female gender, despite presenting lower rates of functional maintenance, were not associated with higher risks of functional decline in the final model. Higher functional decline in older adults that had private health plans, higher education levels and shorter institutionalization times follows the characteristic profile of private NH residents, who are generally institutionalized with a more advanced impairment process [27].

As a limitation of the study, monitoring losses due to deaths and transfers were approximately 26%. This issue was initially predicted (in a sample of older, frail individuals), and to compensate for it, new individuals were incorporated in the second wave of data collection. Residents who were transferred between participating NH, and those who returned home but were re-admitted to participating NH, continued to be evaluated.

The specific evaluation of functional capacity is recognizably difficult, as occasionally the main caregiver interviewed at the beginning of the study was not present in successive waves. In some cases, differences were noticed between the evaluations of the caregivers regarding classification of the resident (degree of dependency in each activity). Several measures were taken to control this issue: a) only the main researcher applied the functionality gauging instrument; b) previous evaluations were always considered when re-evaluating functional capacity; c) the same caregiver was interviewed in all waves, whenever possible, or a recommended caregiver who had been working at the institution for at least 6 months, and d) second opinions were required when incongruencies were detected in functional capacity dynamics.

Pfeiffer's test for evaluation of cognitive state is still not validated in Brazil, but it was selected because the currently validated instruments (with higher cognitive demands), would have caused a floor effect in the classification of residents, characterized by high frequencies of frailty and cognitive impairments. Another aspect that must be mentioned is that chronic diseases could have been under-registered, due to under-diagnosis of these conditions in NHs. To compensate for this issue, health professionals also were interviewed to gather the maximum amount of information, and disease registration was improved in the successive evaluation waves.

The sample herein considered is representative of the elderly institutionalized population of the studied city, thanks to the participation of most NHs of the city that was demonstrated in the calculation of the sample size. It must be highlighted that there were no data losses throughout successive waves, regarding functional capacity evaluation in the older adults initially registered in each institution. Although it could happen that the older adult was not present at the NH at the moment of data collection, information was provided by the nursing assistant. For independent variables, the majority of older adults presented complete data; for the remainder of the sample, missing data was negligible (under 15%).

With the aim of facilitating international comparison, the interest variable was evaluated by an instrument based on previous studies on functional dynamics in institutionalized older adults. This study also stands out due to the wide variety of considered variables, including nutritional and medication information, obtained thanks to the multidisciplinary character of the research project. To the best of the authors' knowledge, this is the first survival analysis on functional decline carried out in institutionalized older adults in Latin America. Due to the design characteristics of the longitudinal study itself, it is possible to establish causal relationships between independent variables and the analyzed outcome, which facilitates the practical implications of this study.

It can be concluded that the probability of suffering functional decline after 2 years was approximately 56% in this sample of institutionalized older adults. The survival curve for maintenance of functional capacity presents an exponential form, indicating that there is a more pronounced initial decline that is smoothed out afterwards. The prognostic factors for functional decline were initial severe cognitive impairment as well as continence decline and incidence of hospitalizations. These conditions are susceptible of prevention and treatment and, therefore, this study highlights the importance of intervening in these aspects with the objective of delaying the onset of impairment processes.
